# Genome sequence and annotation of the B3 mycobacteriophage Phayeta

**DOI:** 10.1128/MRA.00915-23

**Published:** 2023-11-28

**Authors:** Emily Bishop, Warren Earley, Alexandra Greco, Emma Hofseth, Emma Kinerson, Brandon Lafayette, Nestor Llanot-Arocho, Brittney Mazen, Megan Cevasco, Daniel C. Williams

**Affiliations:** 1 Department of Biology, Coastal Carolina University, Conway, South Carolina, USA; Queens College, Queens, New York, USA

**Keywords:** Bacteriophage

## Abstract

Mycobacteriophage Phayeta was extracted from soil near Myrtle Beach, South Carolina using *Mycobacterium smegmatis* as a host. Annotation of the 68,700 base-pair circularly permuted genome identified 104 predicted protein-encoding genes, 34 of which have functional assignments.

## ANNOUNCEMENT

**Fig 1:**
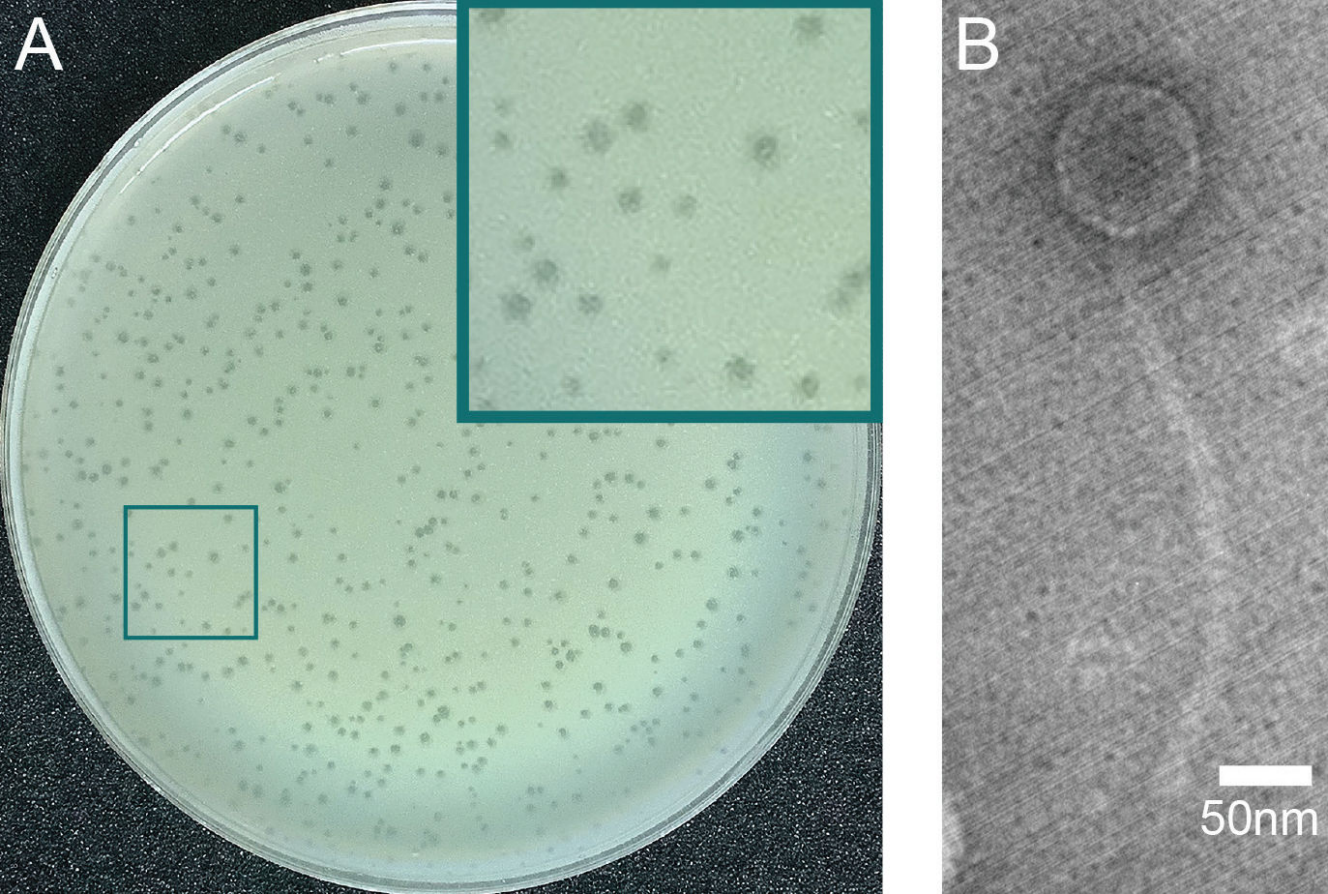
The mycobacteriophage Phayeta. (**A**) Plaque assay. Purified samples of Phayeta (10 ul) were mixed with 250 ul of saturated *M. smegmatis* MC^2^155, then combined with 3 mL of molten 7H9 top-agar and poured on 90 mm plates. Incubation at 37°C for 48 hours resulted in the production of clear small plaques. (**B**) Transmission electron microscopy of Phayeta negatively stained with 1% uranyl acetate and imaged using a JEOL JEM 1230 at 100 kV.

*Mycobacterium smegmatis* is a genetically tractable model of *Mycobacterium tuberculosis* and phages that infect *M. smegmatis* have the potential to be therapeutically useful (1, 2). Mycobacteriophage Phayeta was directly isolated from a soil sample taken near Myrtle Beach, South Carolina (Global Positioning Coordinates 33.75493N, 78.90047 W), using standard protocols (3). The soil sample was washed in 7H9 liquid media, filtered (0.22 um), and 500 uL of the filtrate mixed with 250 mL of saturated *M. smegmatis* mc^2^155 for 15 minutes before being plated in top agar and incubated at 37°C for 48 hours. Phayeta produced small clear plaques that are characteristic of non-temperate phages (Fig. 1A). Plaque size was measured with ImageJ and ranged from 0.5 mm to 1.7 mm with an average of 1.0 ± 0.2 mm (mean ± SD, *n* = 50) (4, 5). Negative-staining transmission electron microscopy of Phayeta (Fig. 1B) revealed a siphovirus morphology consisting of an isometric capsid (diameter = 76.6 ± 3.6 nm) and a long flexible tail (274.7 ± 11.6, *n* = 9 viral particles).

Phayeta DNA was extracted from high-titer lysates using the Wizard DNA Clean-Up Kit (Promega) and analyzed by agarose gel electrophoresis. Intact DNA was prepared with the Ultra II Library Kit (New England Biolabs) and sequenced on the Illumina MiSeq platform (v3 reagents). A total of 446,915 single-end 150 base reads provided 278× coverage of the Phayeta genome. Sequence reads were assembled and verified using Newbler (v2.9) and Consed (v29) (6). The genome is 68,700 base-pairs long, has 67.5% GC content, and contains circularly permuted genomic ends. Comparison of gene nucleotide similarity of phages within the Actinobacteriophage Database (phagesdb.org), placed Phayeta in subcluster B3 (7). Among this cluster, Phayeta shares the greatest gene content similarity with Casbah (94.6%) and Kronus (91.7%).

Initial draft annotation of Phayeta was done using the GenMark (v2.5), Glimmer (v3.02), and ARAGORN algorithms within DNA Master (v5.23.56) using the bacterial, archaeal, and plant plastid code (8–10). Subsequent manual annotations employed Phamerator (v7), Starterator (v1.3), and PECAAN (11). Both PhagesDB and NCBI nonredundant databases were searched for sequence-based homology comparison via BLAST (7, 12). Predicted protein structure comparisons used HHPRED (v3.18) to search the PDB mmCIF70, Pfam-A, and NCBI Conserved Domain databases using default parameters. A total of 104 protein-encoding genes were identified, 34 of which have a functional assignment.

Most genes on the left arm of Phayeta encode structural or assembly proteins with the right arm containing genes involved in DNA replication. Consistent with a non-temperate lifestyle, Phayeta lacks genes encoding an identifiable integrase or repressor. Both Lysin A and Lysin B are present. Although homology searching failed to identify a holin-encoding gene, a small cluster of transmembrane-segment-containing genes (gp17-gp20) have sequence and syntenic similarities with genes of *Gordonia* phages that are a predicted holin cassette (13). There are five minor tail proteins, including gp33 which shows full-length similarity to Corofin gp33. Both these phages have a single nucleotide indel polymorphism in this gene but at different locations suggesting distinct mutational events.

## Data Availability

Phayeta is available at GenBank with Accession No. OR159655 and Sequence Read Archive No. SRX20630268.
